# Pediatric Physical Medicine and Rehabilitation

**DOI:** 10.3390/children9070954

**Published:** 2022-06-25

**Authors:** Min Cheol Chang

**Affiliations:** Department of Physical Medicine and Rehabilitation, College of Medicine, Yeungnam University, Taegu 38541, Korea; wheel633@gmail.com; Tel.: +82-53-620-4682

Pediatric rehabilitation medicine is a discipline that enables children with acquired or congenital disabilities to reach their maximum physical, mental, social, occupational, and educational potential [1]. Pediatric rehabilitation fundamentally differs from adult rehabilitation in that it involves treating the disabilities that develop between birth and adulthood, while the goal of rehabilitation for adults is to help restore the lost function due to a disease or injury. Since childhood is a stage of active growth and development, children present with more comorbidities than adults. The rehabilitation of children with disabilities involves more complex diagnostic and treatment modalities than that of adults. Thus, it is a specialized field that requires adequately equipped healthcare workers [2]. The pediatric rehabilitation team should have sufficient knowledge to accurately assess the functional injuries and provide the optimal treatment modalities in order to allow children to reach their full potential.

In this Special Issue, seven clinically significant publications are discussed [3,4,5,6,7,8,9]. Song et al. [6] documented the neurodevelopmental outcomes of infants who underwent surgery for congenital heart disease for 2 years. More than half of the patients had developmental delays, emphasizing the need for early screening and rehabilitation. Chen et al. [3] investigated the development of impulse control behavior in children. Their results help analyze the behavioral performance levels and impulse control functions associated with various developmental disorders. Kwon et al. [4] showed that the combination of botulinum toxin A and extracorporeal shock wave therapy effectively managed spasticity in children with spastic cerebral palsy. Yun et al. [9] reported the usefulness of repeated urimal tests of articulation and phonation in diagnosing childhood apraxia of speech. Pastor-Pons et al. [5] reported a correlation between transportation using a baby pushchair and increased severity of positional plagiocephaly. A randomized controlled trial by Sung et al. [7] demonstrated the therapeutic potential of touch-screen-based cognitive training in children with severe cognitive impairment. Yu et al. [8] found that children with sensory processing disorders had limited supination and pronation of the foot complex as well as reduced ankle pronation, which assists the push-off and toe grip movements.

As the editor responsible for this Special Issue, I am elated to contribute to the current body of knowledge on pediatric rehabilitation and to present the novel treatment discussed in these seven studies. I hope that this Special Issue will further advance pediatric rehabilitation research.
